# Mesoporous-silica nanofluidic channels for quick enrichment/extraction of trace pesticide molecules

**DOI:** 10.1038/srep17171

**Published:** 2015-11-24

**Authors:** Pengcheng Xu, Chuanzhao Chen, Xinxin Li

**Affiliations:** 1 State Key Lab of Transducer Technology, Shanghai Institute of Microsystem and Information Technology, Chinese Academy of Sciences, 865 Changning Road, Shanghai 200050, China

## Abstract

As nanofluidic channels, uniaxially oriented mesoporous-silica is, for the first time, *in-situ* self-assembled in a microfluidic chip for quick enrichment/extraction of ng L^−1^(ppt)-level organo-phosphorous (OP) pesticide residue from aqueous solution to ethanol. This micro/nano combined pre-treatment chip is essential for following gas chromatography-mass spectrometry (GC-MS) quantitative analysis. Featuring huge surface area and dense silanol groups at the inwall surface, the mesoporous-silica is uniaxially self-assembled in a micro-reservoir to form a pile of nanofluidic channels (diameter = 2.1 nm). The captured/enriched pesticide molecules in the nanochannels can be efficiently extracted by much smaller volume of ethanol due to its much higher solubility to OP. In our affirming experiment, three mixed OP pesticides of dichlorvos, paraoxon and chlorpyrifos (in water) are captured/enriched by the nano-channels and eluted/extracted by only 0.6 mL ethanol. The whole process only takes 16 min. The GC-MS quantitative results for the extracted three pesticides indicate that the extraction recovery achieves 80%. The achieved limit of quantification (LOQ) and the limit of detection (LOD) are 100 ng L^−1^ and 30 ng L^−1^, respectively. The nanofluidic-channel pre-treatment technique is promising in various application fields like agriculture and food safety security.

With the rapid development of lab-on-a-chip and micro-total-analysis system (μ-TAS) technologies^1^,^2^, microfluidic chips are becoming enabling tools for pre-treatment of ultra-low concentration and trace amount analytes^3^. In such trace-sample pre-treatment applications like high-efficiency enrichment and extraction, surface-to-volume ratio of the fluidic channel is a key factor. Compared to the conventional microfluidic channel, the analyte molecules in solution tend to interact with the surface groups at the nanochannel wall, since the analyte molecules are confined in the tiny spaces of the nanochannels. Therefore, various pre-treatment chips have been recently developed that are constructed with nanofluidic channels^4^,^5^,^6^,^7^,^8^,^9^,^10^. In order to form nanofluidic channels, top-down fabrication methods have been tried by either mask-less sculpting with focused ion beam (FIB) or masked dry-etching with electron beam (EB) lithography. Such top-down fabrication generally suffers high fabrication cost and low throughput. Since the top-down methods can only form one layer of nano-channel in the microfluidic channel, they are not suitable for pre-treating samples at high rate. Anodic aluminum oxide (AAO) was ever employed for the formation of nano-channels. Unfortunately, the technique suffers non-uniformity of channel diameter and difficulties in surface chemical modification. On the other hand, bottom-up self-assembly has been developed rapidly that is herein considered a suitable method to build densely stacked nanofluidic channels with uniform diameter for quickly liquid-sample treatments.

Featuring densely packed multiple-nanopores, high pore volume, uniform pore-size and tunable surface chemical properties by inwall modification of functional molecule-layer, mesoporous-silica^11^,^12^,^13^ (especially with well organized pore orientation^14^,^15^,^16^) is considered as an ideal candidate for constructing the nanofluidic channels. However, in the previous studies, such kind of techniques was mainly used by developing some nanoscale size-effect of the nano-channels for proton transportation^17^,^18^,^19^,^20^. For fluidic lab-on-chip applications, however, highly dense nanochannel thick-pack should be regioselectively integrated into a microfluidic channel, where the whole fluidic cross section should be fully filled with the highly oriented nanochannels. It is really a technical challenge.

To the best knowledge of the authors, mesoporous-silica nano-channel fluidic chip is herein, for the first time, proposed and developed for trace sample quick treatment prior to gas chromatography-mass spectrometry (GC-MS) analysis. In this study, the mesoporous-silica nano-channels are regioselectively self-assembled in a micro-reservoir to form a nanofluidic chip. Then, the mesoporous-silica nanofluidic channels are proposed for quick enrichment/extraction of trace-amount organo-phosphorous (OP) pesticide from aqueous solution to ethanol, which is a typical water-miscible solvent. The enrichment/extraction function is based on the interaction of silanol-group with P = O(S) and the following elution by ethanol. After the pre-treatment, the following GC-MS analysis indicates that the fast enrichment and high-efficiency water-to-ethanol extraction of ppb-level pesticides have been achieved successfully.

## Results

### Characterization of the nanofluidic chip

**Figure 1:**
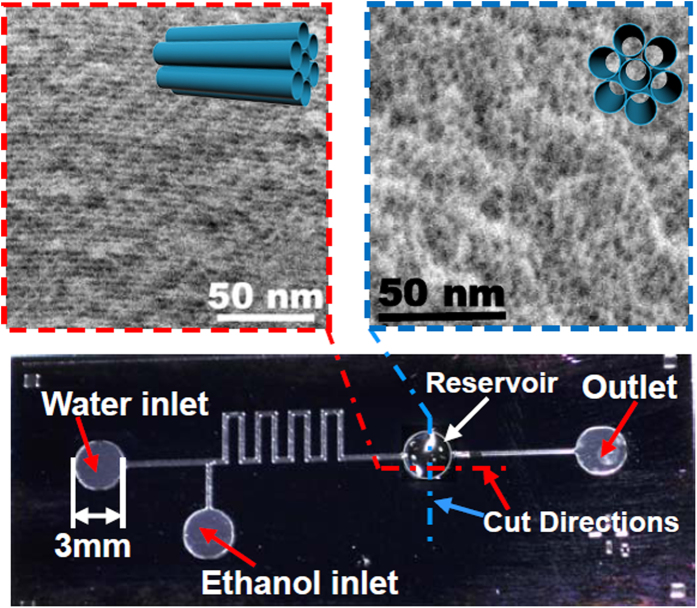
Top-view photograph of the extraction/enrichment microfluidic chip. The mesoporous-silica nano-channels in the reservoir are characterized by high-resolution SEM. The two cross-sectional SEM images are with the sample cut along the two directions denoted in the figure. The SEM at the left-side shows the longitudinal direction of the nano-channels and the right one shows the transverse direction of the channels where the nano-pore entrances can be seen.

The chip is fabricated with top-down silicon micromachining technology as well as bottom-up self-assembly of mesoporous silica. Shown in Fig. 1, the extraction reservoir is at the center of the micromachining-formed silicon micro-channel, with the diameter as 3 mm and depth as 20 μm (i.e. the reservoir volume is about 1.4 × 10^−4^ mL). The mesoporous-silica nano-channels are regio-selectively grown in the reservoir by using evaporation induced self-assembly (EISA) process. According to our previous research^21^, the mesoporous silica tends to form uniform film onto the silicon substrate that was pre-modified with 3-aminopropyltriethoxysilane (APTES). The phenomenon is explained as follows. Based on “like dissolves like” theory, there must be an affinity between the APTES monolayer and the nano-material precursor which is mainly composed by ethanol and the amine-group contained CTAB (cetyltrimethylammonium bromide) template. Thus, the nano-material precursor can be locally coated at the APTES modified micro-region. At the neighboring regions however, super-amphiphobic SAM of FAS-17 (1H,1H,2H,2H-perfluorodecyltrichlorosilane) is pre-modified for preventing the precursor from overflow, thereby restricting the area of the formed nano-channels. High-resolution scanning electron microscopy (SEM) images inset in Fig. 1 show the direction and entrance morphology of the densely stacked mesoporous-silica nano-channels. According to the images for many fabricated samples, in the confined space^22^,^23^ of the SAM pre-functionalized reservoir, it is found that the nano-channel stack is with preferred orientation always along the micro-channel direction. In other words, the inlet and the outlet of the nano-channels are always exposed to the fluidic path of the micro-channel. The spontaneously orientational formation of the nano-channels is possibly due to following reason. The nano-channels are constructed via EISA, where ethanol solvent in the precursor prefers to evaporate into ventilate open space. Among the in-plane directions in the reservoir, the ethanol more easily vaporizes along the longitudinal direction of the micro-channel to reach the open spaces at the double sides of the reservoir. The preferred orientation of the nano-channel formation along the channel direction speeds up evaporation of the internal solvent in the precursor that facilitates fast formation of the nano-channel stack. In order to close the nanofluidic channels and lead out the inlet/outlet pipes, a polydimethylsioxane (PDMS) capping plate was bonded with the silicon fluidic chip.

N_2_ sorption experiment further indicates that pore diameter of the nanofluidic channels is approximate 2.1 nm (see online Supplementary Figure S1). By using Brunauer-Emmett-Teller (BET) method, the specific surface area of the mesoporous-silica nano-channels was measured as 339 m^2^ g^−1^. Compared with those mesoporous silica particles where the area of outer surface can be quite large, the whole film containing the nano-channels has negligible outer surface area. In addition, the tiny amount sample of the nano-channels makes difficulties in the BET measurement that can cause weighting inaccuracy.

The characteristic-peak located at 3740 cm^−1^ in Fourier transform-infrared spectrum (FT-IR) pattern can be assigned to vibration of the ≡Si-OH group that indicates abundant silanol groups existing at the inwall surface of the nano-channels (see online Supplementary Figure S2). The amount of silanol (≡Si-OH) groups in the nanofludic channels is further obtained by using thermogravimetric analysis (TGA). The TGA measurement was conducted under N_2_ atmosphere, since -OH groups tend to dehydrate along with temperature rising at inert atmosphere. In the obtained TGA curve, there is a weight-loss ratio of 2.4wt% in the range of 150 °C to 850 °C (see Supplementary Figure S3 online). Based on the experimental data including the BET surface area and TGA result, the density of ≡Si-OH group *a* can be calculated according to the equation of 24


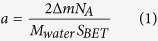


where Δ*m* is the weight-loss ratio, *N*_*A*_ is the Avogadro constant, *M*_*water*_ is the molecular weight of H_2_O and *S*_*BET*_ represents the BET surface area. For the density of ≡Si-OH group, *a* is calculated as 4.8 nm^−2^, which is similar to the reported value of 4.9 nm^−2 ^25.

### Fluidic phenomena of the chip

It is known that the inwall interface of the mesoporous-silica nano-pores features good wetting property to polar molecules like water. Since the interface covered with dense ≡Si-OH groups exhibits hydrophilic characteristic^26^, the contact-angle *θ* of water on the silica surface should be less than 90°. According to Young-Laplace pressure expression of


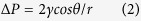


where *γ* is surface tension and *r* is radii of curvature (*r* < 0). Herein the Δ*P* value should be negative under the condition of 0 < *θ* < 90° 26,27,28. Due to the negative Δ*P*, water or ethanol can be driven into and spontaneously flows through the mesoporous-silica nanofluidic channels, without additional force such as external electrical field or high pressure. This advantage brings in simplicity of chip construction and operation convenience. As shown in online Supplementary Figures S4a and S4b, the polar solution flows smoothly through the nanofluidic channels. In contrast, as is shown in Supplementary Figures S4c, S4d and the Movie S1, the non-polar solvent of n-hexane (or toluene) cannot flow into the nano-channels and, under extra-high driving pressure, the pre-bonded PDMS capping plate turns to be broken into crack by the blocked n-hexane solution. Therefore, dissolved in water, the target analyte molecules in trace-level sample can be introduced into silica-based nanofluidic channels for pre-treatment.

**Figure 2:**
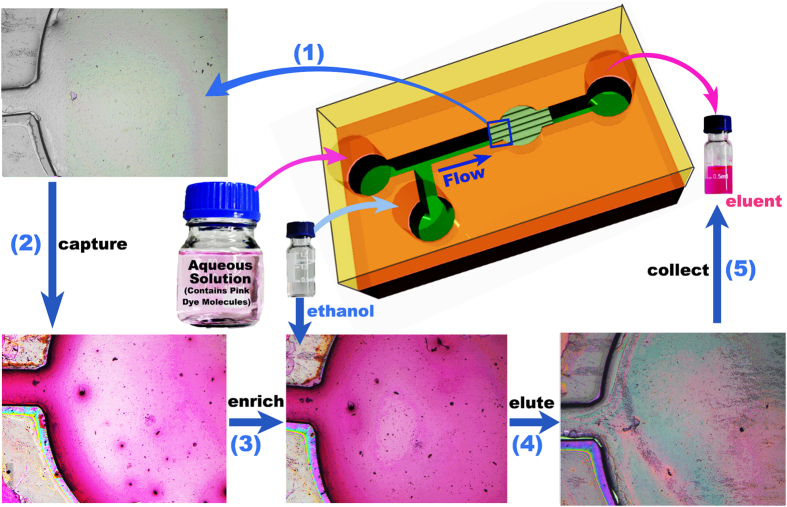
Experiment of acid-fuchsin pink dye concentrated and micro-extracted from water to ethanol, by using the nano-channel integrated micro-chip. The photograph of the glass bottle was taken by P.C.X.

In order to validate the enrichment/extraction function of the nano-channels, serial experiments are performed. To visualize the extraction process in the nano-channel integrated fluidic-chip, an experiment is implemented with the steps shown in Fig. 2 as: (1) The left-part of the reservoir can be seen in the photo-micrograph, where the nano-channel stack is accommodated. (2) The pale-pink colored fuchsin-dye aqueous solution is flowed through the nano-channels. With the interaction between ≡Si-OH group of the nano-channels and -NH_2_ group of the fuchsin molecules, the fuchsin molecules are captured at the inner walls of the nano-channels. (3) The color of the nano-channels turns deeper and deeper along with continual flow of the dye solution, indicating enrichment of the dye molecules. (4)-(5) Thereafter, ethanol flows through the nano-channels. With strong solubility of ethanol to fuchsin, the color at the nano-channels becomes to fade and the color of the ethanol solution at the outlet turns to deeper and deeper. The results well confirm the feasibility of the pre-concentrating/extracting function with the nano-channels.

### Enriching/extraction results for trace pesticides molecules

Associated with health of human-beings, food safety becomes a hot research topic and, in more serious cases, causes social unrest^29^,^30^,^31^,^32^. Great efforts from both research institutions and government departments have been made on developing various effective techniques to secure food safety^3^,^33^,^34^,^35^,^36^,^37^,^38^. Related to farm products, food-safety accidents are often induced by pesticide residues and illegal usage of veterinary drugs^31^,^39^,^40^. Among the various chemical contaminate sources of farm products, residual agricultural chemicals like organo-phosphorus (OP) pesticides are very toxic and harmful to health^32^, thereby being convinced as significant analytes to be detected. Nowadays, GC-MS is used for identification and analysis of trace-level OP chemicals due to its competitive cost and satisfied qualitative/quantitative analysis capability^40^,^41^. Unfortunately, such residual pesticide samples are normally aqueous solutions that are collected from plants or soil. It is worth noting that the aqueous characteristic of the samples cannot be directly detected by GC-MS, as GC-MS operation strictly requires the analyte to be pre-dissolved in an organic solvent (e.g., ethanol) instead of water. The reason lies in that the solvent of water may cause: *i*) the stationary phase on the GC-column inner-wall will be washed away; *ii*) the vacuum system and the electron-impact ionization would be damaged and; *iii*) the MS filament may be burn out. Prior to GC-MS analysis, the sample has to be previously extracted into a GC-MS compatible organic solvent. However, the widely used liquid-liquid extraction (LLE)^42^,^43^ is herein not favorable, since the conventional organic solvent like benzene or chloroform is harmful to health and environmentally unfriendly^44^. Unluckily, the environmentally benign organic solvents like ethanol are normally inter-soluble with water. Although solid-phase extraction (SPE) has been rapidly developed recently^45^,^46^,^47^, the SPE method generally needs a large amount of sample, as well as, it is labor intensive and time consuming. On the other hand, pre-enrichment is often needed to improve the analytical resolution, as the original concentration of the pesticide residue is normally as low as ppb level. When low-cost micro-fluidic chips are used for sample pre-treatment, mL or even sub-mL level sampling volume is needed to realize on-site pre-treatment and quick detection for the applications like inspection of pesticide residue for public food-safety security. Due to all the above-mentioned reasons, a novel feasible enrichment/extraction route is highly in demand for quick detection of trace amount of OP pesticides.

**Figure 3:**
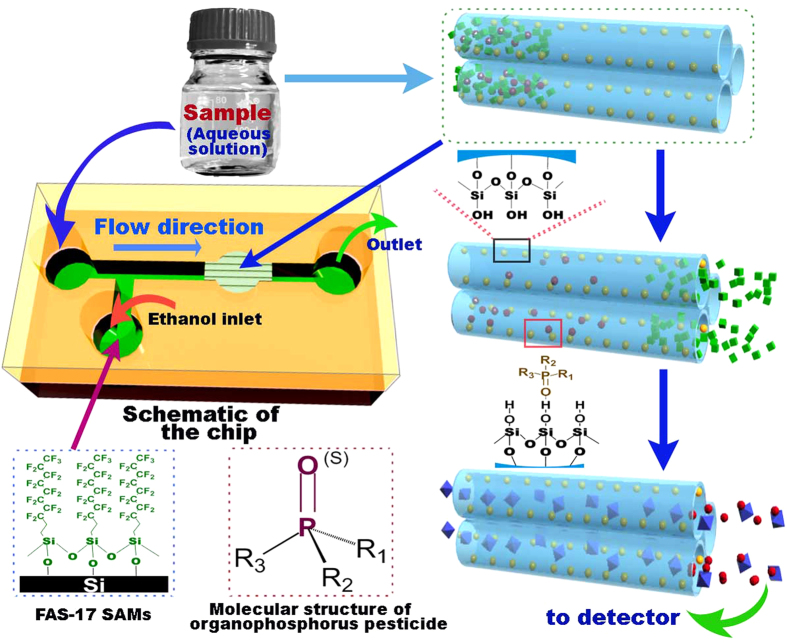
Schematic procedure for pre-extracting/enriching trace OP pesticide from aqueous solution to a water-miscible organic solvent. The photograph of the glass bottle was taken by P.C.X.

Benefited from the confined space of the nanofluidic channels, chemical interaction between the target analyte molecules and the ≡Si-OH groups in the nano-channels can be dramatically enhanced^48^. In the nanofluidic channels, the OP target molecules can be quickly captured and effectively enriched by the ≡Si-OH groups on the inwalls of the nanofluidic channels via specific adsorption with the P=O (or P=S) groups of the OP molecules^49^,^50^,^51^,^52^,^53^. With the sample continually flowing through the nano-channels, the trapped OP molecules can be pre-concentrated. Assuming that one ≡Si-OH group captures one OP molecule, we can estimate the OP-molecule loading capacity per gram material as 1.6 × 10^21^ (i.e., 2.7 mmol). The calculation is based on the measured BET surface area (339 m^2^ g^−1^) and the known ≡Si-OH density on the silica surface (4.8 ≡Si-OH groups/nm^2^)^25^. Thereafter, the trapped molecules can be eluted from the nano-channel surface and extracted by desired polar organic solvents (like ethanol) that feature much higher solubility to the molecules. After the fast lab-on-chip pre-treatment to the residual pesticide sample, the enriched/extracted product can be quantitatively analyzed by GC-MS. The pre-concentration of the sample helps to improve limit of detection (LOD) of the original sample in trace-level water solution. Figure 3 shows the extraction/enrichment process, where densely stacked nano-channels are integrated in a micro-reservoir of a microfluidic chip.

**Figure 4:**
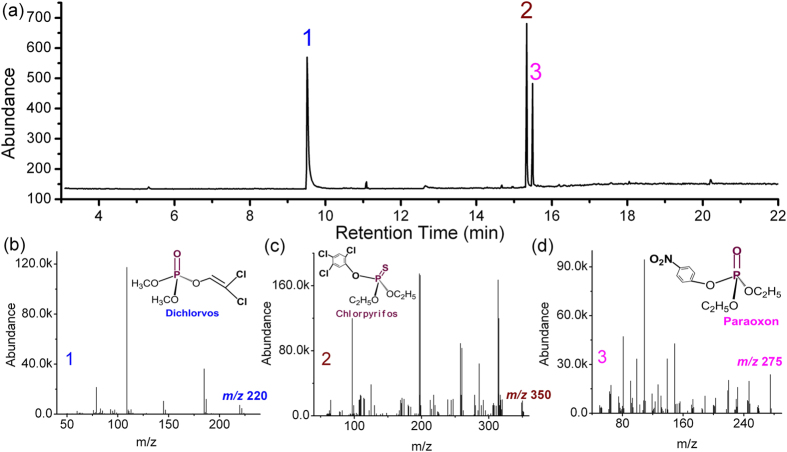
GC-MS analysis for the extracted ethanol solution, which contains three kinds of organophosphorous pesticides of dichlorvos, paraoxon and chlorpyrifos. (**a**) GC-MS obtained total ion current chromatogram of the extract. (**b**–**d**) Mass spectra for the compounds of 1, 2 and 3 in Fig. 4a, respectively. According to the identified results with the NIST08 mass spectral library, the compounds of 1, 2 and 3 are clearly assigned to dichlorvos, chlorpyrifos and paraoxon, respectively.

According to the regulation of World Health Organization (WHO), the allowable aqueous concentrations of the three pesticides are given as: 20 μg L^−1^ for dichlorvos^54^, 30 μg L^−1^ for chlorpyrifos^55^, 10 μg L^−1^ for parathion which can be metabolized to paraoxon^56^, respectively. Hence, an aqueous solution containing the three kinds of mixed OP pesticides of dichlorvos, paraoxon and chlorpyrifos (all with 15 μg L^−1^ concentration, diluted from standard pesticide stock solution) is firstly prepared (detailed in Supplementary information online). Thereafter, small amount (10 mL) of the sample solution is introduced into and then flowed through the nano-channels for molecular capturing and pre-concentration. Normally, OP compounds have much higher solubility in ethanol than in water, e.g., the solubility of chlorpyrifos is only 2 mg kg^−1^ in water but reaches to 500 g kg^−1^ in ethanol. Based on the great disparity in solubility, the OP molecules pre-captured in the nano-channels are effectively extracted by ethanol solution of a much smaller volume of 0.6 mL (compared to the original sample volume of 10 mL). The whole pre-treatment is completed within 16 min. The following GC-MS analysis results in Fig. 4 reveal that all the three pesticides can be clearly detected from the ethanol extract. Attributed to the strong enrichment function of the nano-channels, the method could be used to pre-treat the original aqueous OP sample with concentration of lower than 15 μg L^−1^ (i.e. 15 ppb). In contrast with the mL-level adsorbent volume used in conventional solid-phase extraction (SPE) technology, herein the nano-channel stack in the reservoir is only 1.4 × 10^−4^ mL in volume, which is advantageous in faster pre-treatment to trace amount of sample. In addition, the ultra-small volume of the nano-channels facilitates to recondition and recover for repeated uses. After the chip is used for 120 cycles, the waste ethanol (used for the last-time reconditioning) is analyzed by the selected ion monitoring (SIM) mode of GC-MS, resulting in that no OP molecule can be identified in the waste ethanol. The next-cycle pre-treatment with the same chip indicates no obvious deterioration in extraction performance. Besides, the micro-chip can be low-cost batch fabricated by silicon micromachining technology and, thereby allowing for disposable use. Instead of the bulk SPE instrument, the nano-material integrated fluidic lab-chip is competent for fast and low-cost extraction/enrichment of trace sample. Moreover, the nano-channel surface can be modified with varied functional groups for versatile pre-treatment of various chemicals. Featuring the above-mentioned merits, the proposed nano-porous chip shows potential in many applications, especially in on-the-spot rapid detection for public food-safety inspection. For sample concentration/extraction with the low-cost microfluidic chip, small volume sample means short time needed for liquid flowing through the micro/nano channel, thereby realizing rapid pre-treatment. Such rapid method and in-expensive micro-chips are widely demanded.

The huge surface area of the nano-channels (larger than 300 m^2^ g^−1^) and the dense ≡Si-OH surface groups (4.8 ≡Si-OH groups/nm^2^) is helpful for fast and high-efficiency extraction of trace samples. With dichlorvos (DDVP) as example, the extraction recovery (ER) of the nano-channel extractor is evaluated by the GC-MS quantitative analysis results. The characteristic fragment mass ions of 79, 109 and 187 m/z are used for quantification of DDVP in the SIM mode. Shown in Supplementary Figure S5 online, a calibration curve is firstly obtained by plotting the concentration of the standard DDVP ethanol solution versus the peak-area integral. According to the standard curve and the GC peak-area integral value of characteristic ions in Fig. 4, the DDVP extract of 0.6 mL concentration in ethanol is enriched to 200 μg L^−1^. According to the 10 mL volume and 15 μg L^−1^ concentration of the original water solution of DDVP, the ER value of about 80% is obtained by calculation according to the relationship of


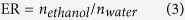


where *n*_*ethanol*_ is the extracted dichlorvos amount in ethanol and *n*_*water*_ is its amount in original water solution. In consideration of the ultra-small sample amount (10 mL), trace concentration (15 ppb) and the difficult water-to-ethanol quick (within 16 min) extraction, so high an achieved extraction-recovery (ER) is acceptable.

Thereafter, in order to measure the LOD and LOQ (limit of quantification) values of our chip, we serially dilute the standard solution until the signal-to-noise ratio (S/N) of the GC-MS response approaches 10 to obtain LOQ and 3 to get LOD. According to our experimental results, the LOQ of our chip is about 100 ng/L and the LOD is about 30 ng/L (the corresponding GC spectra are given as Supplementary Figure S6 and Figure S7 online, respectively). In order to study the influence of inorganic ions, NaCl was added to the standard solutions before sample enrichment/extraction. As is described in the previous reports^57^,^58^, the ion effect of the inorganic ions of Na^+^ and Cl^−^ can reduce the solubility of organic substance like DDVP in water. Thus, in order to enhance the SPE efficiency, NaCl can be added into the aqueous solution before the procedure of SPE. According to our experiment however, almost the same extraction efficiency can be achieved with or without the addition of NaCl. The reason is discussed as follows. On the one hand, the ion effect caused by the addition of NaCl can lower the solubility of DDVP in water, which is helpful for DDVP enrichment. On the other hand however, the Cl^-^ ion can be adsorbed on the nanochannel surface to block the DDVP adsorption, thereby lowering the enrichment efficiency. By counteraction of the two effects, the addition of NaCl has no obvious interference to the DDVP enrichment. Furthermore, to investigate the anti-interfering property of the chip to organic substances, real toxic of 40 wt% DDVP emulsion is herein used for examination. Containing a large amount of organic substances like xylene, the real toxic DDVP emulsion was often used to prepare pesticide solution in agricultural fields. Compared with the results from the standard solution of pure DDVP, the organic substances like xylenes (meta-xylene, para-xylene and ortho-xylene) somewhat bring negative effect on the concentration/extraction of DDVP and deteriorate the LOD from 30 ng L^−1^ to 70 ng L^−1^. The reason lies in that the emulsifier like xylene enhances the solubility of DDVP molecules in water.

For quantitative comparison of extraction performance, a commercial Supelclean™ ENVI-Carb™ SPE (solid phase extraction) tube was used to experimentally extract DDVP. The results showed that the LOD of the SPE tube to DDVP is about 10 μg L^−1^, with the GC spectrum given as the Supplementary Figure S8 online. Our nano-channel device achieved the LOD of 30 ng L^−1^ to DDVP. Compared with our fast sample processing method (within 16 min), the time-consuming SPE method normally takes longer time than 1 hour to complete the process, since it is composed of complex procedures including activation, sampling, washing, drying, elution and concentration. Besides poor LOD and slow processing rate, SPE is solvent consuming (at least 1 mL needed) and cannot be repeatedly used. Compared with the commercial SPE tube, our chip exhibits significant improvement in extraction performance.

Finally, the performance of this method is compared with the existing methods like SPE, SFE (supercritical fluid extraction), SPME (solid phase microextraction) and MSPD (matrix solid phase dispersion), with the results listed in Table 1. Although molecule extraction with SFE method features high sensitivity and low solvent consuming, the method is disadvantageous in expensive equipment that limits its application field. SPME generally features high sensitivity and low solvent consuming. Unfortunately, the durability of the coating at the needle surface is not enough to sustain multiple uses. As for the MSPD method, the reusability is poor since the sorbent has to be mixed with the analyte and the sorbent is difficult to be re-generated for multi-time usage. In contrast, our method features merits in high speed, low cost, high sensitivity and low solvent consuming. Besides, our pre-treatment nanofluidic chip shows potential in on-line sampling of GC-MS.

## Discussion

This study proposes and demonstrates mesoporous-silica nanofluidic channels integrated in a microfluidic pre-treatment chip to quick enrich and extract trace organo-phosphorus (OP) pesticide from aqueous solution into ethanol solvent (for further GC-MS quantitative analysis). With the novel pre-treatment nanofluidc channels, 10 mL aqueous solution of 15 ppb (or μg L^−1^) OP molecules is captured by ≡SiOH groups in the silica nano-channels of huge specific surface area. The trapped and enriched pesticide molecules are then eluted and extracted by only 0.6 mL ethanol of much higher solubility. GC-MS results for the enriched/extracted pesticide mixture (containing three kinds of OPs) indicate that the silica nano-channels can be used to residual pesticides with concentrations lower than 15 μg L^−1^. With DDVP selected as the analyte target, 100 ng L^−1^ LOQ and 30 ng L^−1^ LOD are both achieved by using the method. The experimentally validated enrichment/ extraction function with the mesoporous-silica nano-channels is promising in trace sample pre-treatment lab-chip applications such as agriculture and food-safety detection.

## Methods

### Fabrication of mesoporous-silica nanofluidic channels

The process for preparing the precursor of the mesoporous silica is given as follows. Firstly, 50 mL TEOS (tetraethylorthosilicate) was dissolved in 50 mL ethanol. Under vigorous stirring for 90 min, 4.1 mL H_2_O and 1 μL HCl (12 M) was added into the flask immersed in a water bath of 60 °C. The solution was taken off from the water bath while stirring is kept. After the solution cools down to room temperature, 16.5 mL H_2_O and 76 μL HCl (12 M) were drop-wise added under stirring for 15 min. With stirring for 15 min, the solution was put into the water bath of 50 °C. With the stirring kept at room temperature, the solution was taken off from the water bath. After 250 mL ethanol was added to dilute the solution, 8.4 g CTAB was put into the solution. Finally, clear sol solution was obtained after the CTAB was absolutely dissolved. The molar composition of this sol solution was 1 TEOS: 0.1 CTAB: 23 ethanol: 5 H_2_O: 0.004 HCl. This stock solution was prepared as the precursor to form nano-channels in the following procedure.

**Figure 5:**
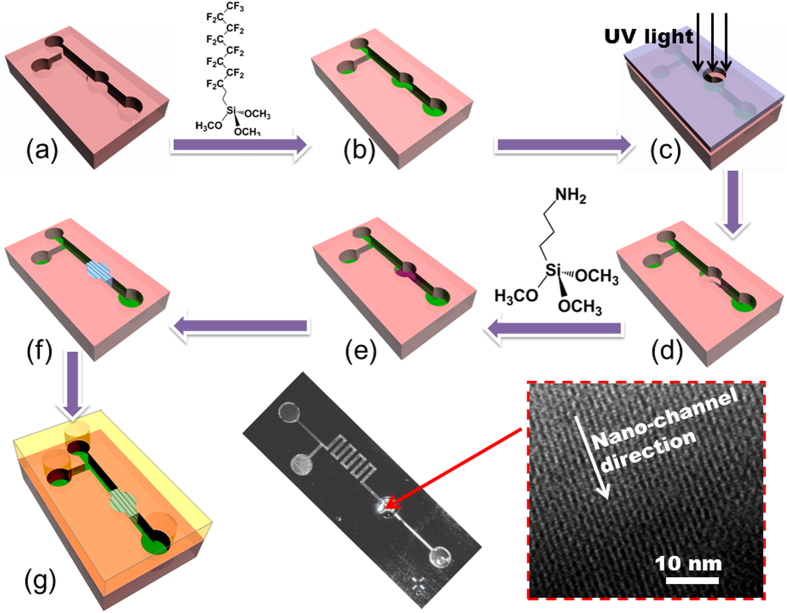
Fabrication process of the nano-channels integrated micro-chip and the optical photograph of the fabricated micro/nano fluidic chip. The transmission electron microscopy (TEM) image clearly shows the highly directional mesoporous-silica nano-channels.

Integration of the nano-pore material into the reservoir of the micro-channel chip is with the process schematically shown in Fig. 5 and described as follows. (a): The micro-channel was wet etched on silicon wafer, with an extraction-reservoir at center. (b): By using molecular vapor deposition (MVD, MVD100E System, Applied Microstructures, Inc.), the micro-channel surface was self-assembled with super-amphiphobic SAM (self-assembled monolayer) of FAS-17. (c) and (d): Through a pre-patterned hard-mask, ultra-violet (UV) light was region-selectively introduced to localized remove the FAS-17 at the reservoir as we described in our previous publication^59^. By using a commercial system (PSD-UV, Novascan Technologies, Ames, IA), region-selective UV-light exposure was introduced to the wanted micro-regions through a hollowed hard-mask. The photolithographic nano-surface patterning was performed at 100 °C for 30 min. During the removing process of FAS-17 SAM, the UV lamp was kept 4 cm above the micro-channel surface. The localized UV-light (wavelength: 185 ~ 254 nm) irradiation generated ozone from environmental oxygen. The generated ozone locally oxidized the –CH_2_– at the root of the molecule-chain to remove the SAM there. After the UV irradiation, an ≡Si–OH covered surface was formed at the micro-regions. (e): -NH_2_ terminated SAM of APTMS was re-grown at the FAS-17 removed region of the reservoir. (f): The nano-channel precursor solution was dropped onto the APTMS-modified reservoir with a pipette. By EISA, mesoporous-silica nano-channels were locally formed on top of the SAM layer of APTMS. (g): The chip was treated for 2 min (at 800 mTorr air) with a plasma cleaner (Harrick Plasma cleaner PDC-002). Then a PDMS cover was plasma treated together with the chip for 1 min. Finally, the micro-chip with the integrated nano-channels was capped by room-temperature bonding with the PDMS cover. It is worthy pointing out that, the CTAB template can be simultaneously oxidized and removed during the plasma treatment.

### Pesticide extraction/enrichment experiment

1.5 mL of the 10 mg L^−1^ standard solution for the pesticide mixture was diluted by distilled water to 1 L and the resultant solution concentration was 15 μg L^−1^. Then, the solution was introduced to the extraction/enrichment microfluidic chip by using a KDS-250 syringe pump (KD-Scientific Inc., Holliston, MA, USA). The experimental process is detailed as follows. Firstly, 10 mL of 15 μg L^−1^ pesticide solution was filled in a plastic syringe. Then, the syringe was placed in the syringe pump to connect to the inlet of the chip via a micro-line tube. The flow rate was set as 1 mL min^−1^ and, thus, the aqueous sample capturing/enrichment procedure last for 10 min. The waste solution was collected at the outlet by using a small vial. After the pesticide solution entirely flows through the chip, clear air was injected into the chip to blowing dry the chip. Then, 0.6 mL absolute ethanol was introduced to the chip, with the flow rate of 100 μL min^−1^, to elute/extract the previously captured pesticide molecules within 6 min. For the subsequently implemented GC-MS identification, the ethanol eluent containing the extracts was collected (at the outlet) into a screw-cap vial (with 2 mL volume, Agilent Technologies Inc., Palo Alto, CA, USA). For repeated usages, the nano-channels in the chip were washed with ethanol (10 mL) for more than three times. To verify the complete desorption of the pesticide molecules, the waste ethanol in the 3rd washing procedure was collected and analyzed by GC-MS.

The composition of the ethanol eluent was identified by using a capillary GC-MS apparatus (Agilent 7890 A-5975C). The model of the commercially available column is DB-17MS (30 m × 0.25 mm × 0.25 μm). The used temperature program was 3 min at 40 °C, a ramp of 15 °C min^−1^ to 280 °C and hold-time of 5 min. 1 μL ethanol eluent was injected into GC with a spilt ratio of 50:1. The flow rate of the helium carrier gas was 1.1 mL min^−1^. MS was operated in electron impact mode with the source temperature as 250 °C, quadrupole temperature as 150 °C and ionizing voltage as 70 eV. The scan was performed in the range of m/z 50 to 500. The composition of the eluent was identified by using a NIST08 mass spectral library.

## Additional Information

**How to cite this article**: Xu, P. *et al.* Mesoporous-silica nanofluidic channels for quick enrichment/extraction of trace pesticide molecules. *Sci. Rep.* **5**, 17171; doi: 10.1038/srep17171 (2015).

## Supplementary Material

Supplementary Information

## Figures and Tables

**Table 1:** Performance comparison between current work and the existing methods.

Parameters	Typical existing methods				This work
	SPE	SFE	SPME	MSPD	
processing period of time	>1 hr	>30 min	>1 hr	<30 min	<20 min
reusability	poor (single time)	good (many times)	poor (a few times)	poor (single time)	good (many times)
cost	low	high	low	low	low
sensitivity	low	high	high	high	high
solvent consuming	high (several mL)	low	low (sub-mL)	low (sub-mL)	low (sub-mL)
on-line sampling	not	not	not	not	yes
