# Supplementary material for: Mesoporous-silica nanofluidic channels for quick enrichment/extraction of trace pesticide molecules
Source: Sci Rep. 2015 Nov 24;5:17171. doi: 10.1038/srep17171 (PMC4657006; doi:10.1038/srep17171)
Supplement: Supplementary Information [file srep17171-s1.doc]

**Supplementary information**

Mesoporous-silica nanofluidic channels for quick enrichment/extraction of trace pesticide molecules

Pengcheng Xu, Chuanzhao Chen, and Xinxin Li*

State Key Laboratory of Transducer Technology, Shanghai Institute of Microsystem and Information Technology, Chinese Academy of Sciences, Shanghai 200050, China

**Experimental**

**Chemicals.** Pesticide sample of dichlorvos, paraoxon and chlorpyrifos (all in analytical grade) were purchased from Supelco (Bellefonte, PA, USA). Cetyltrimethylammonium bromide (CTAB) was purchased from Sigma-Aldrich. APTES (3-aminopropyltriethoxysilane) and FAS-17 (1H,1H,2H,2H-Perfluorodecyltrichlorosilane) were purchased from Gelest (Morrisville, PA, USA). Acid fuchsin dye, tetraethylorthosilicate (TEOS), ethanol, 37% HCl, 30% H_2_O_2_, 98% H_2_SO_4_ were of analytical grade and purchased from Shanghai Chemical Corp. Polydimethylsioxane (PDMS) was obtained from Dow Corning (Midland, MI, USA).

**Preparation of pesticide standard solution.** Dichlorvos, paraoxon and chlorpyrifos were selected as target pesticides in this research. For preparing the standard solution of the pesticide mixture, the three kinds of pesticides, with each weighting 10 mg, were sequentially added into an amber-colored volumetric flask (1000 mL). Then, about 100 mL of distilled water was added to dissolve the pesticides. Distilled water was thereafter added until the liquid level reached the volume-mark of 1000 mL. The prepared standard pesticide stock solution, with 10 mg L^-1^ (i.e. 10 ppm) concentration for each kind of pesticide, was further diluted to the desired concentrations for the following extraction/enrichment experiment. For quantitative analysis using GC-MS, three series of standard solution with various concentrations of 20-300 μg L^-1^ (or ppb) were prepared for the three kinds of pesticides. Herein ethanol was used as solvent.

**Characterization.** High resolution scanning electron microscopy (HR-SEM) and transmission electron microscopy (TEM) images of the nanoporous samples were taken using an FEI Magellan 400 XHR ultrahigh resolution cold field emission scanning electron microscope and a JEOL-2010F TEM apparatus. Nitrogen sorption isotherms were measured at 77 K by using a Micromeritics ASAP 2020M system. Specific surface area and pore size distribution were calculated using Brunauer-Emmett-Teller (BET) and Barrett-Joyner-Halenda (BJH) methods. Fourier transform-infrared spectrum (FT-IR) experiment was performed with a Bruker Vertex 70v infrared spectrometer (under the vacuum <100Pa). Optical images and videos were carried out on a Leica DM4000 microscopy.

**Fluidic experiment.** Serial experiments are performed to validate the extraction/enrichment function of the nanofluidic channels integrated micro-channel chip. As are shown in Figures. S1a, b, water (known as polar solvent) solution of acid fuchsin dye (showing bright pink color in aqueous solution) can smoothly flow through the nano-channels. With increase of the flowing period of time, the pink color in the reservoir becomes darker and darker that indicates effective capturing of the nana-channels to the dye molecules. In contrast, as are shown in Figures S1c, d, non-polar organic solvent (n-hexane here) cannot flow through the nano-channels that indicate fluidics selectivity of the nano-channels. The experimental results preliminarily imply that the silica based nanofluidic channels can be used to concentrate analytes and extract them from a polar solvent like aqueous solution into another polar solvent like ethanol, even though the two polar solvents may completely miscible with each other. The experimental phenomenon of the oil (*n*-hexane) fluid blocked by the nano-channels is recorded in Movie S1.


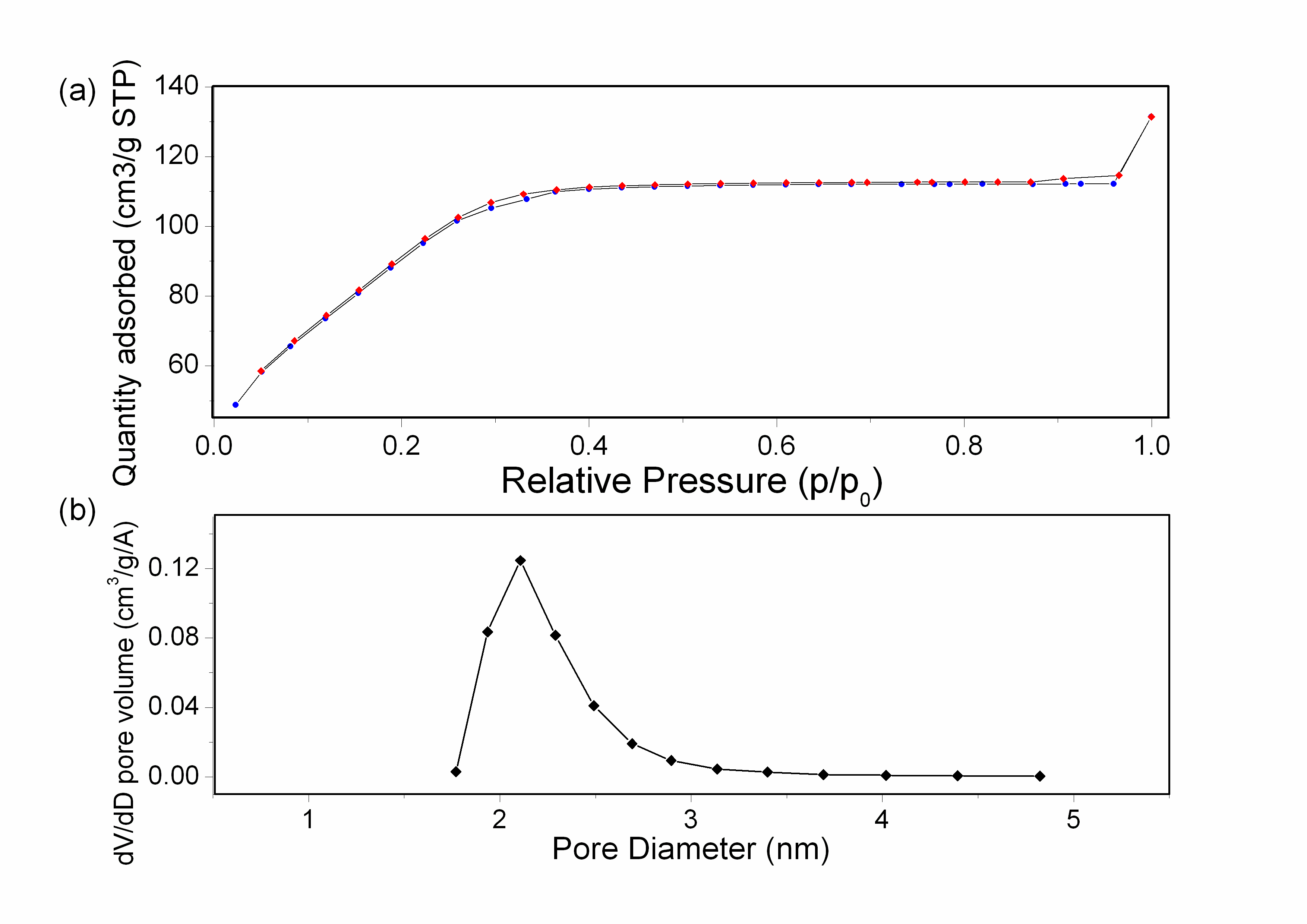


**Figure S1.** (a) Nitrogen sorption isotherm and (b) pore size distribution of the mesoporous-silica sample that is calculated according to BJH theory. According to standard N_2_ sorption measurement, the specific surface of the as-prepared mesoporous-silica-monolith is measure as 339 m^2^ g^-1^, the pore volume is 0.2 cm^3^ g^-1^ and the average pore size is 2.1 nm.


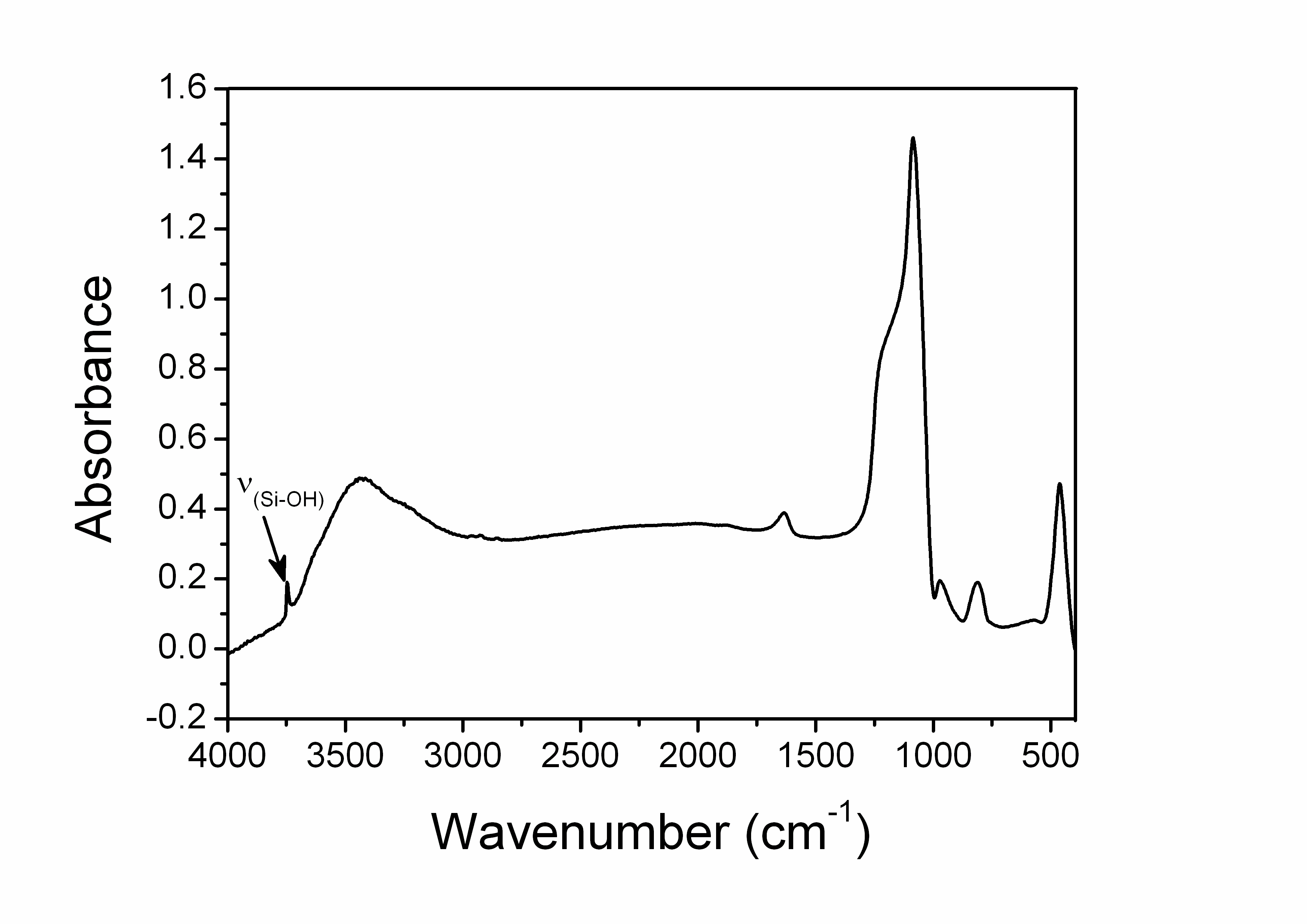


**Figure S2.** FT-IR characterization of the mesoporous-silica nano-channel sample. The absorbance peak located at 3740 cm^-1^ can be assigned to the abundantly existing ≡Si-OH groups at the nano-channel surface.


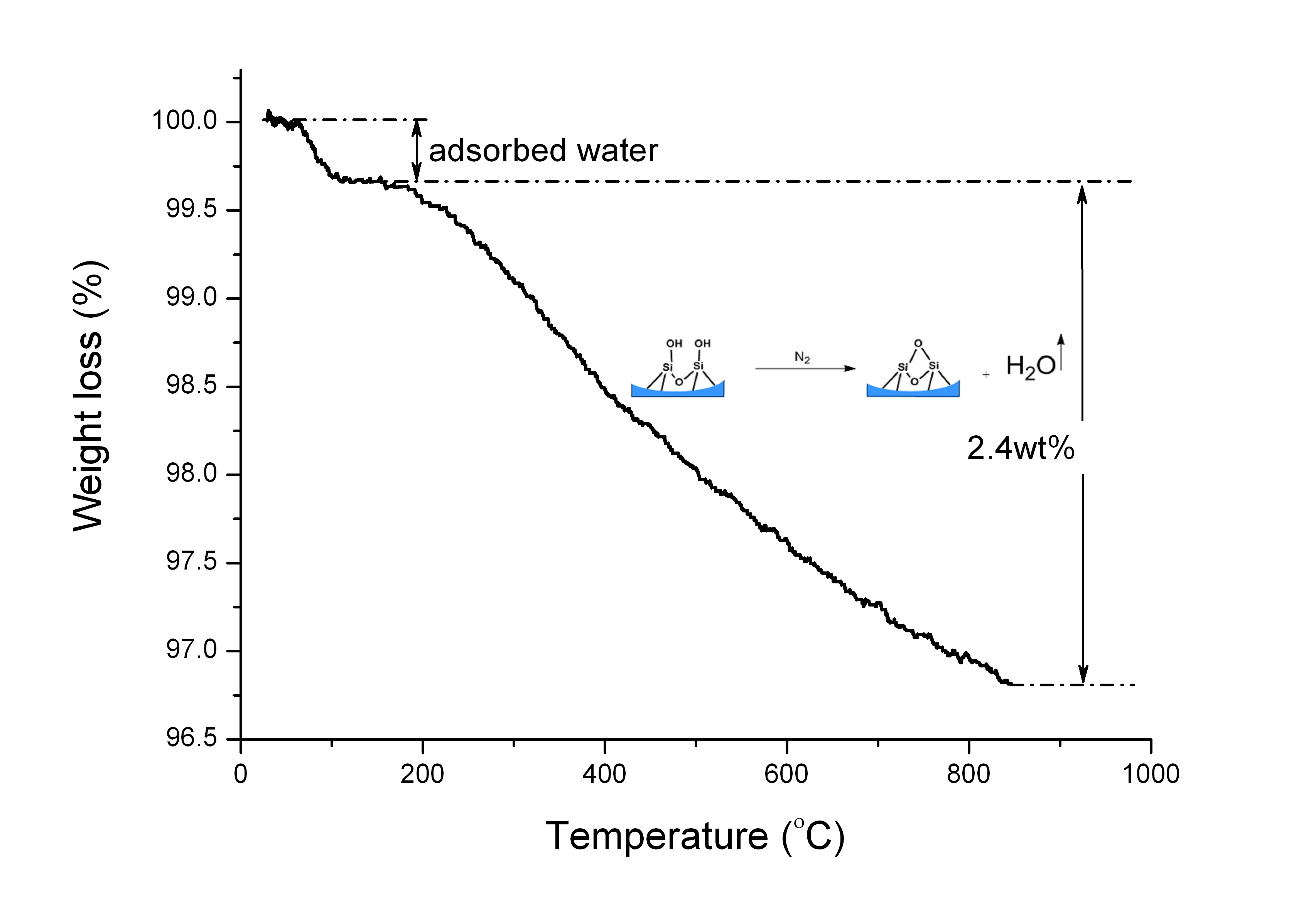


**Figure S3.** TGA curve of the mesoporous-silica nano-channel sample under N_2_ atmosphere, which was obtained by using a NETZSCH STA 449 F3 simultaneous thermal analyzer. The experiment was performed from 25°C to 850°C, with a heating rate of 10°C/min.


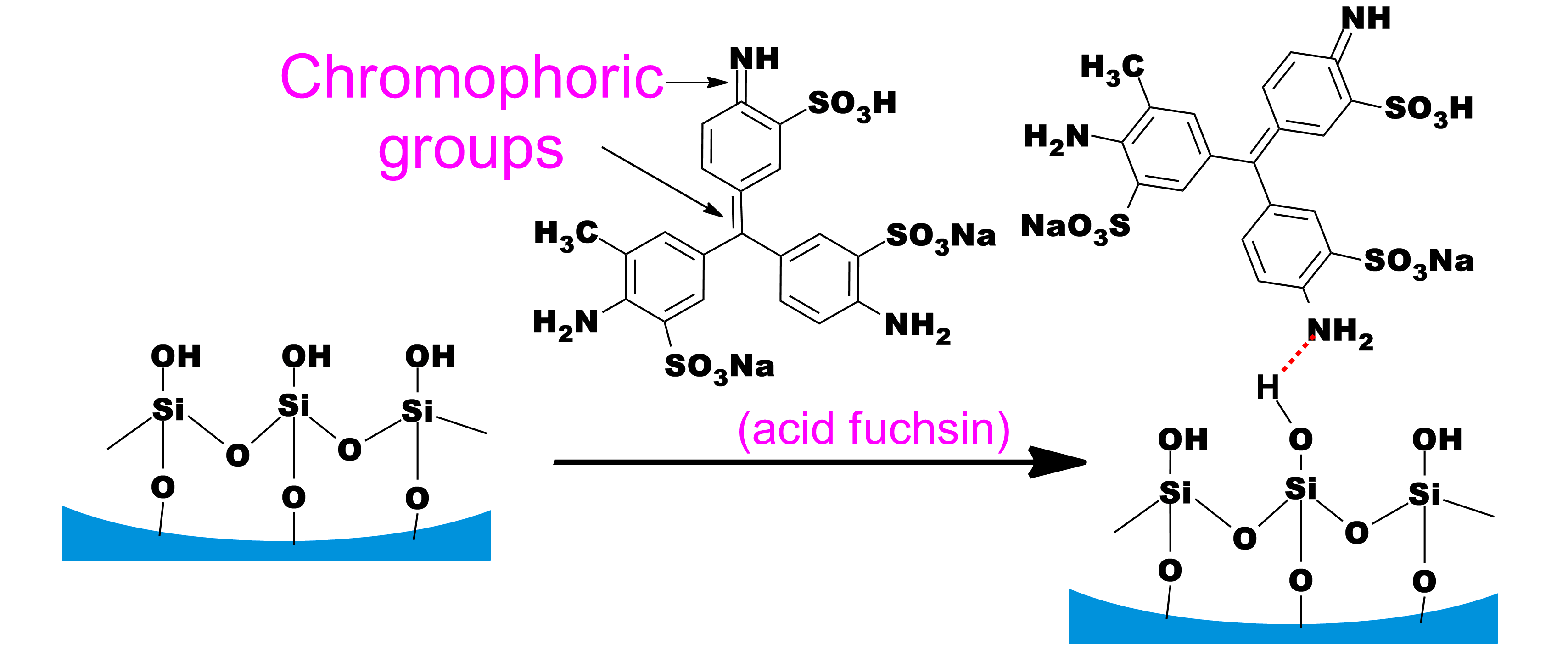

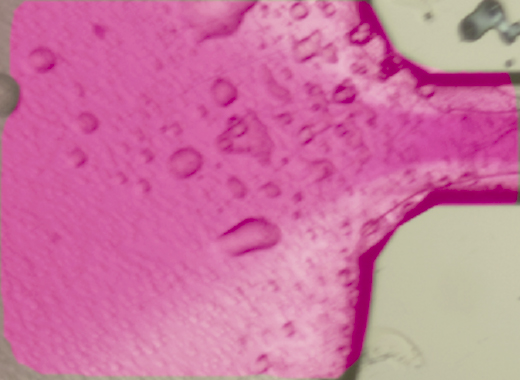


(a)


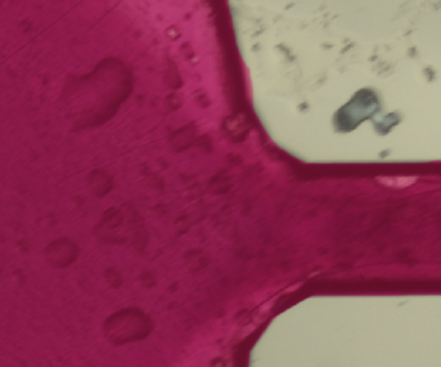


(b)


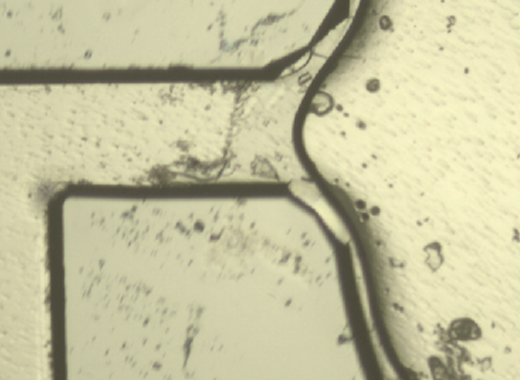


(c)


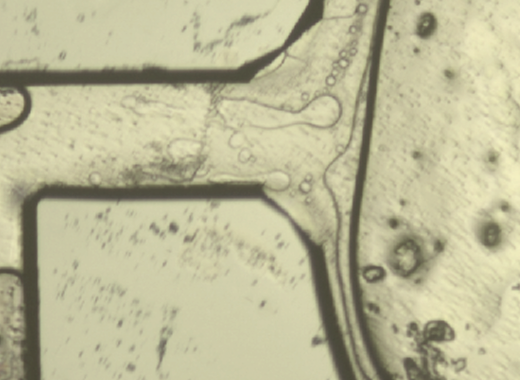


(d)

**Inlet of water**

**solution**


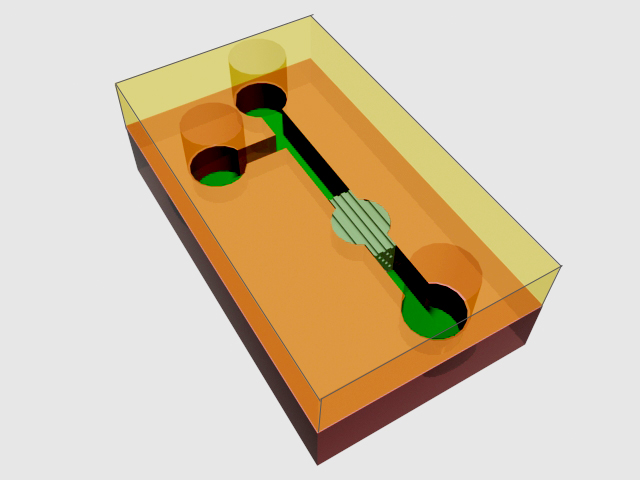


**Inlet of oil**

**Outlet**

**Figure S4.** Water solution of pink-colored dye and oil (*n*-hexane) are sequentially used to test the fluid-selective properties of the nano-channels. The capturing mechanism of the fuchsin molecules by the silanol-groups on the nano-channel walls are denoted at the bottom.


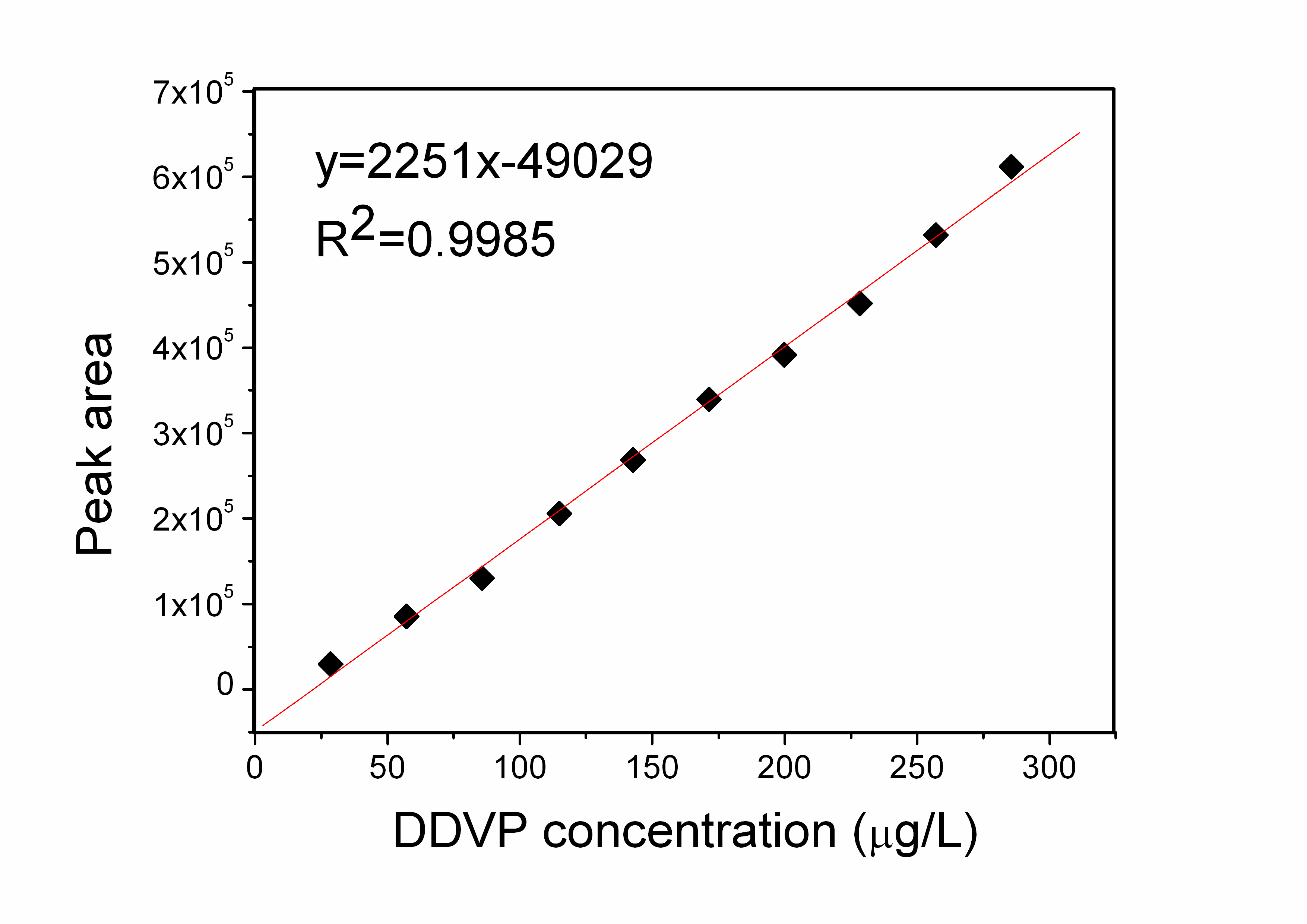


**Figure S5**. Calibration curve of the concentration of DDVP in ethanol in terms of the peak-area integral of the characteristic fragment mass ions is plotted for GC-MS quantitative analysis. Based on the results in Figure 4 in the text, the peak area integral is obtained as 4×10^5^. Accordingly, the concentration of the enriched/extracted DDVP ethanol solution is derived as 200 μg L^-1^.


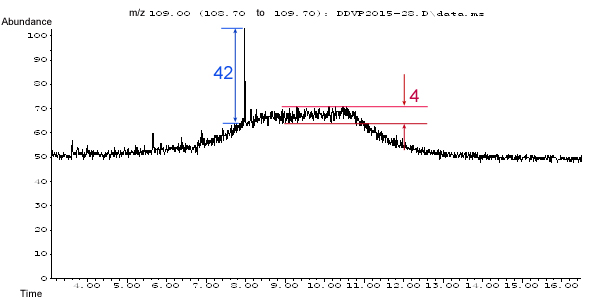


**Figure S6.** GC spectrum of extracted DDVP, which was pre-treated from 100ng L^-1^ DDVP aqueous solution by using the chip. Based on the regularity of signal-to-noise ratio (S/N) >10, the LOQ of our method is about 100ng L^-1^.


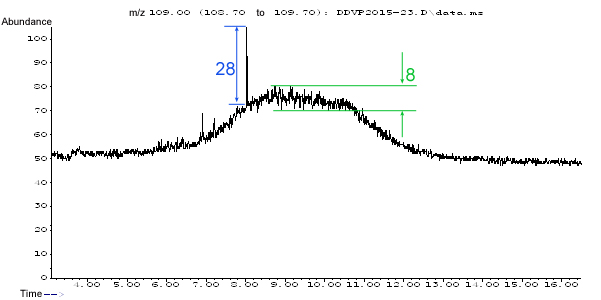


**Figure S7.** GC spectrum of the extracted DDVP, which was pre-treated from 30ng L^-1^ DDVP aqueous solution by using the chip. Based on the regularity of signal-to-noise ratio (S/N) >3, the LOD of our method is estimated as 30ng L^-1^.


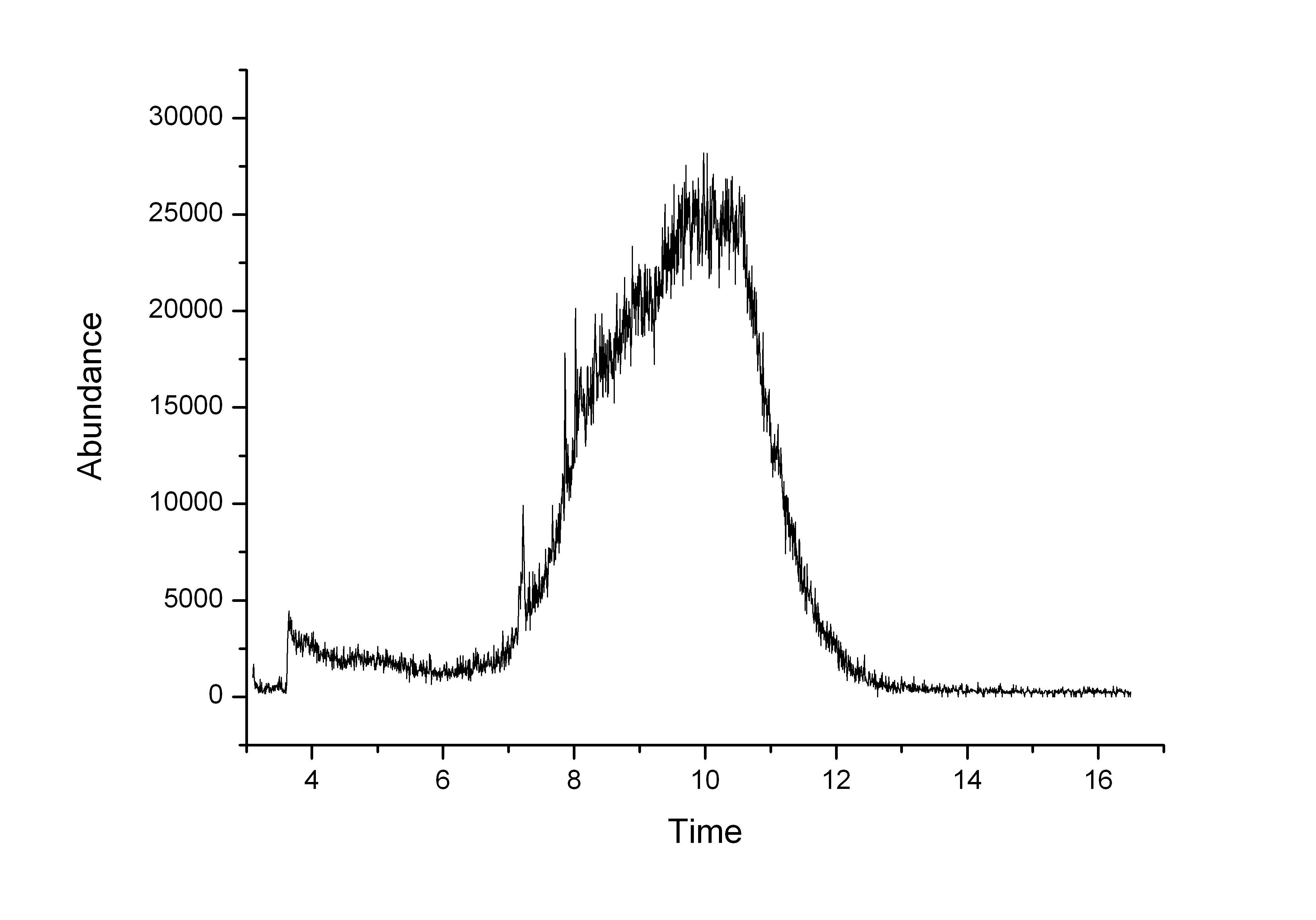


**Figure S8.** GC spectrum of the extracted DDVP, which was pre-treated from 10μg L^-1^ DDVP aqueous solution by using the commercial Supelclean™ ENVI-Carb™ SPE tube. Based on the regularity of signal-to-noise ratio (S/N) >3, the LOD of our method is about 10μg L^-1^.


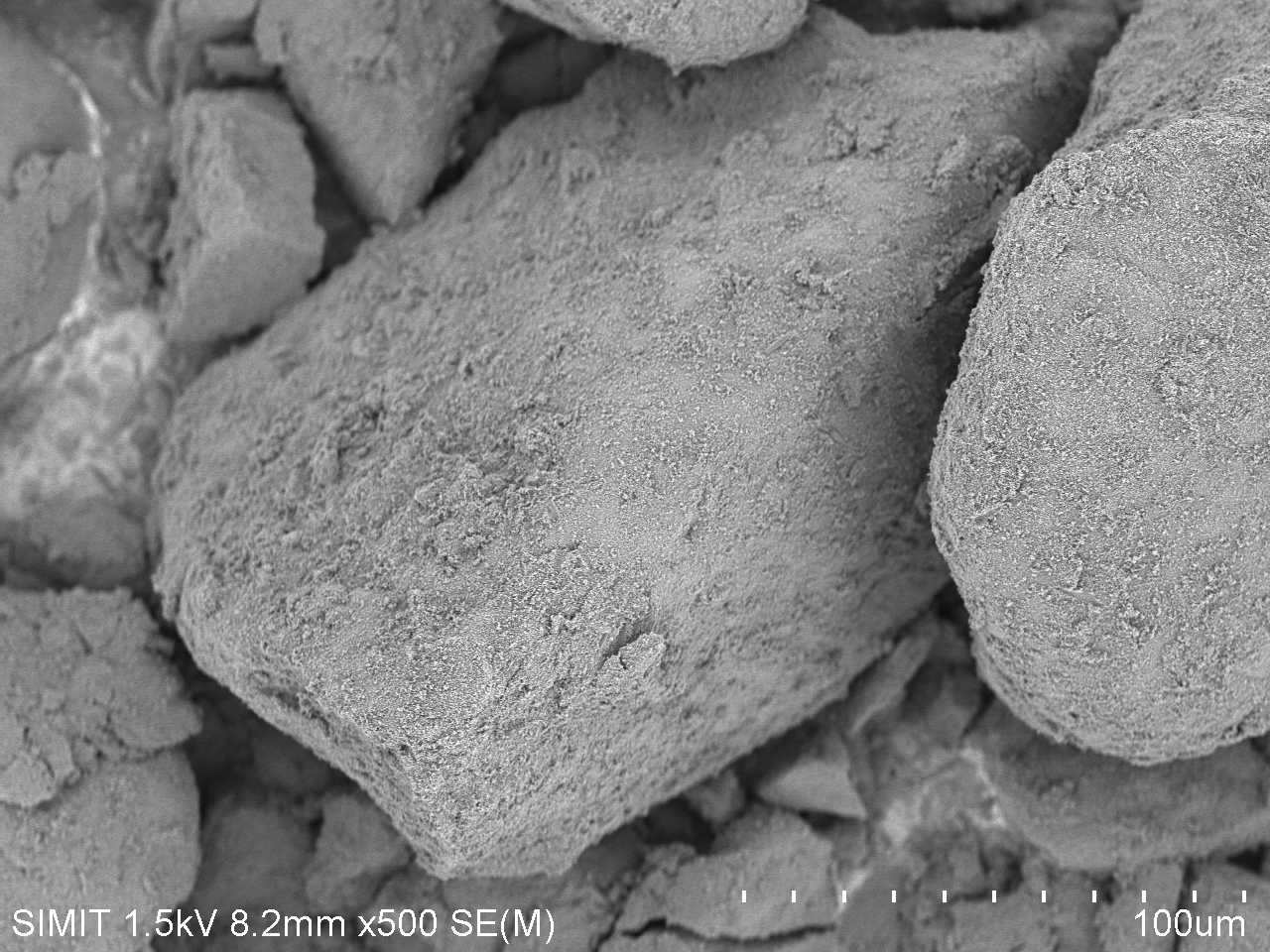

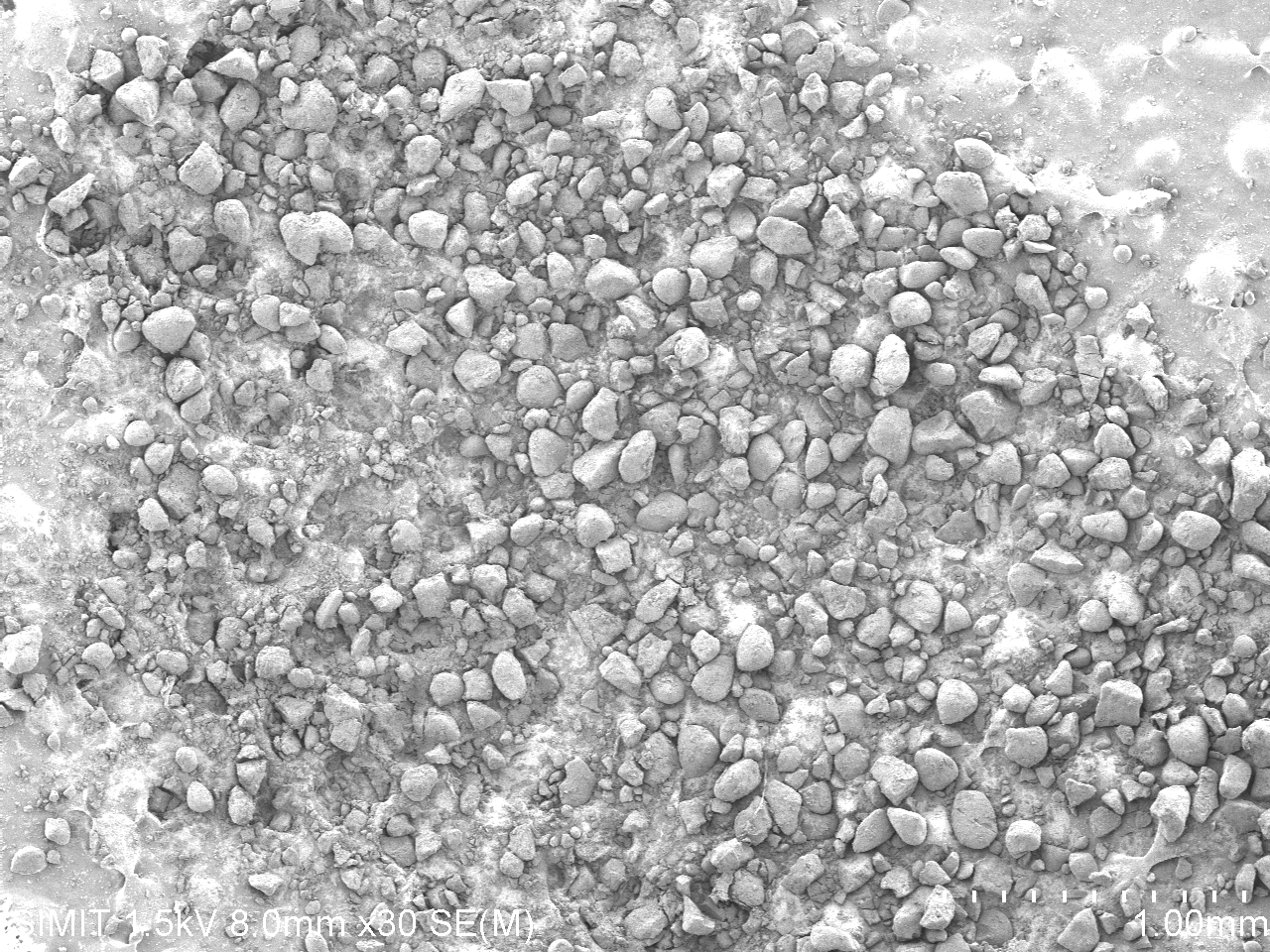


**Figure S9.** SEM images of the graphitized carbon stuffed in the commercial Supelclean™ ENVI-Carb™ SPE tube. According to the SEM images, the graphitized non-porous carbon in the SPE tube was with the inhomogeneous particle size ranged from tens of nanometer to hundreds of micron. The BET surface area of the graphitized carbon is measured as 96.7m^2^/g.
